# PrEP Cascade and Barriers Among Serodifferent Couples in Rural Tanzania: A Prospective Study on Awareness, Uptake, Adherence, and Retention

**DOI:** 10.1007/s10461-025-04957-8

**Published:** 2025-11-21

**Authors:** Anna Eichenberger, Lilian Moshi, James Okuma, Fiona Vanobberghen, Aloyce Sambuta, Olivia Kitau, Leila S. Matoy, Elizabeth Senkoro, Namvua Kimera, Mohamed Mbaruku, Jamali Siru, Raphael Magnolini, Tracy R. Glass, Maja Weisser

**Affiliations:** 1 Department of Infectious Diseases, Inselspital, Bern University Hospital, Universtiy of Bern, Bern, Switzerland; 2 Ifakara Health Institute, Ifakara, Tanzania; 3 St. Francis Referral Hospital, Ifakara, Tanzania; 4 Swiss Tropical and Public Health Institute, Allschwil, Switzerland; 5 University of Basel, Basel, Switzerland; 6 Division of Infectious Diseases, University Hospital Basel, Basel, Switzerland; 7 Arud Centre for Addiction Medicine, Zurich, Switzerland; 8 Institute of Primary Care (IHAMZ), University of Zurich and University Hospital Zurich, Zurich, Switzerland; 9 Kilimanjaro Christian Medical Centre, Moshi, Tanzania

**Keywords:** HIV, Pre-exposure prophylaxis, Serodifferent couples, Prevention, Stigma

## Abstract

Global HIV incidence remains high, particularly in sub-Saharan Africa. Pre-Exposure Prophylaxis (PrEP) prevents HIV transmission in serodifferent couples until the partner with HIV achieves viral suppression on antiretroviral treatment (ART). This study assessed awareness, uptake, and feasibility of a short, 4 month PrEP course alongside the roll-out of dolutegravir-based ART in rural Tanzania. We included serodifferent couples (≥ 15 years), of whom the partner with HIV enrolled in the Kilombero and Ulanga Antiretroviral Cohort (KIULARCO) and initiated ART between September 2020 and September 2021. Partners without HIV with normal kidney function (eGFR > 60 ml/min) and negative HbsAg were offered PrEP. Both partners completed questionnaires on sexual behavior and adherence. Partners without HIV were tested monthly for HIV. Couples were followed until the partner living with HIV achieved viral suppression (< 200 copies/ml). Among 346 people newly diagnosed with HIV, 184 (53%) reported a sexual partner, 97 (53%) partners were tested and 40 (41%) were HIV negative. PrEP awareness was low (9%). Of 37 eligible seronegative partners, 22 (59%) initiated PrEP, and 9 (41%) completed follow-up. No serious adverse events or HIV seroconversions occurred. 21/22 (96%) of PWH were virally suppressed after 3 months. In our study, partner testing, acceptance and PrEP uptake were low. A 4-month course of PrEP was safe and effectively prevented HIV transmission. These findings highlight gaps in the PrEP care cascade in rural sub-Sahara Africa. Lack of awareness and disclosure of HIV status remain significant barriers to PrEP uptake and HIV prevention among serodifferent couples.

**Supplementary Information:**

The online version contains supplementary material available at 10.1007/s10461-025-04957-8.

## Introduction

Despite significant global progress over recent decades in slowing the AIDS epidemic, the number of new HIV infections remains high. In 2023, 1.3 million new HIV infections occurred globally, with more than half of new infections reported from sub-Sahara Africa (SSA) [1, 2]. A steep decline in the numbers of new infections is needed in order to achieve the ambitious target of fewer than 370,000 new HIV infections by 2025, as set by the UNAIDS [2, 3].

Pre-exposure prophylaxis (PrEP) with daily oral PrEP containing tenofovir disoproxil fumarate (TDF) and emtricitabine (FTC) is safe and efficacious in preventing HIV transmission [4, 5]. Since 2015, WHO recommends offering PrEP to all populations at substantial HIV risk, defined as HIV incidence greater than 3 per 100 person–years [6]. With the newly published data on long-acting injectables as PrEP, the options for HIV prevention will become even more effective [7].

While PrEP should be made available to all people at high risk of acquisition, access and uptake remains slow in many countries [8]. Challenges in PrEP rollout include lack in awareness, demand and continuation of PrEP, all of which require better understanding to expand its delivery [9]. To monitor the success and identify the challenges of PrEP programs, a PrEP care continuum has been developed, consisting of PrEP eligibility, PrEP initiation, adherence and retention in PrEP programs [10].

Serodifferent couples remain a significant contribution to new HIV infections in SSA [11], making them a priority population for PrEP delivery [12]. A large study in Kenya and Uganda showed high uptake of PrEP in partners with a seronegative status in the period before the partners with a seropositive status reaches viral suppression, resulting in near elimination of HIV-1 transmission, and showing how PrEP can ensure the stability of a relationship in serodifferent couples [13, 14]. Current guidelines recommend PrEP for partners without HIV until the partner living with HIV achieves viral suppression, using it as a “bridging period” [15]. PrEP can be stopped 28 days after the last risk exposure, thus 28 days after viral suppression in the partner living with HIV. Since WHO recommends the first VL testing after 6 months on ART, PrEP in serodifferent couples is recommended for 7 months. Studies have shown that nearly half of the partners discontinue PrEP earlier [16]. With the roll-out of dolutegravir (DTG)-based ART, viral suppression is achieved more rapidly compared to non-nucleoside reserve transcriptase inhibitor (NNRTI)-based ART [17, 18]. Therefore, the bridging period of PrEP could potentially be shortened.

With this study, we aimed to assess the PrEP care continuum among serodifferent couples in rural Tanzania. Additionally, we aimed to analyze if a shorter bridging period of 4 months is feasible.

## Methods

### Study Design and Setting

A prospective implementation study was conducted at the Chronic Diseases Clinic of Ifakara, the care and treatment centre for people living with HIV (PWH) at the Saint Francis Referral Hospital in rural Tanzania. At enrollment into care all PWH are offered participation in the Kilombero and Ulanga antiretroviral cohort (KIULARCO), a prospective cohort of PWH established in 2005. The cohort collects comprehensive demographic and clinical data, including ART use and monitoring, co-morbidities and treatment outcomes, with details described previously [19, 20].

### Study Population and Procedures

For this study, all people newly diagnosed with HIV aged ≥ 15 years enrolled into KIULARCO with written informed consent from September 2020 to September 2021 with a partner with a seronegative status or partner of unknown status were eligible to answer the baseline PrEP questionnaire including information on sexual behaviour, knowledge about HIV transmission, awareness of PrEP and acceptance of the partner to start PrEP (Supplement 1). In addition, they were asked to bring their partner for testing. If the partner tested negative, and the couple was sexually active with the intention to remain a couple and the newly diagnosed partner living with HIV had initiated ART, the partner without HIV was eligible for PrEP. Exclusion criteria for the partner without HIV included renal impairment (eGFR < 60 ml/min), positive HBsAg, signs of acute HIV infection or being under 18 years and still in school. Partners without HIV received counseling on HIV, transmission risks, and prevention methods. If they agreed to participate and after signing the informed consent form, they completed a questionnaire on sexual behavior, knowledge about HIV transmission, awareness of PrEP, and reasons for choosing to start PrEP (supplement 2). Due to a reluctance of participants to answer questions about sexual risk behavior the baseline questionnaires were amended halfway into the study by changing the order of questions and adding a question about living in the same household, length of relationship and having children together.

The ART regimen for newly diagnosed PWH was a fixed-dose combination of tenofovir disoproxil fumarate 300 mg, lamivudine 300 mg, and dolutegravir 50 mg; PrEP for the partners without HIV consisted of tenofovir disoproxil fumarate 300 mg and emtricitabine 200 mg, both as recommended by Tanzanian guidelines [21].

Follow-up visits were conducted for both partners at 1, 2, 3, 4, and 6 months. If a scheduled visit was missed, a study nurse called the participants within a week and if unsuccessful, contacted a community health care worker (as is also done in routine care at the clinic) to follow up on the client. Lost to follow up was defined as being more than 60 days late for the next scheduled visits for PrEP care. During these visits, sexual behavior, contraceptive methods, symptoms of sexually transmitted diseases, adherence to PrEP and ART, and, if applicable, reasons for PrEP discontinuation were assessed (Supplement 3 and 4). For partners without HIV, HIV rapid tests were repeated at every visit; creatinine and syphilis testing were performed at baseline and after 6 months. In the seropositive partner, HIV viral load was measured at 3 and 6 months. If the viral load was suppressed (defined as < 200 copies/ml), PrEP was discontinued 28 days later. The 200 copies/ml cut-off was based on studies indicating no risk for HIV transmission through unprotected sex for viral loads below this level [22, 23]. In cases showing a viral load ≥ 200 copies/ml; PrEP was continued, and—as part of routine practice—a repeat viral load measurement was conducted after 3 months and after enhanced adherence counselling. Data collection for PrEP ended after 9 months.

### Outcomes

Primary outcome was the proportion of partners without HIV of newly diagnosed PWH who initiated PrEP. Secondary outcomes were the PrEP care continuum including the proportion of newly diagnosed PWH with a sexual partner at risk for contracting HIV (partner without HIV or not tested), the proportion of partners eligible for PrEP and their linkage to PrEP care, defined as attending at least one visit at the clinic for PrEP counselling. Further outcomes were adherence on PrEP, retention in care, side effects, changes in sexual behavior and incidence of HIV infection among partners taking PrEP. For those who newly seroconverted, the study assessed possible drug resistance mutations. The proportion of viral suppression among people living with HIV after 3 months on ART was also evaluated.

Other secondary outcomes were the knowledge of HIV transmission and awareness of PrEP among all newly diagnosed PWH and their partners, as well as reasons for declining PrEP, assessed among PWH with an eligible partner.

### Laboratory Measurements

HIV testing was performed by using a rapid test (Bioline) and, if positive, a confirmatory test was done with a different rapid test (Unigold), according to the National guidelines [21]. Viral load measurement was done using the Abbott Real-time m2000 HIV-1 Assay (Abbott Laboratories, Chicago, Illinois), with a reported range of 40–10,000,000 copies/mL for blood plasma. Hepatitis B and syphilis testing was done using rapid tests (HBsAg and VDRL), and creatinine was measured by Cobas c111 Chemistry Analyzer (Roche Diagnostics, Rotkreuz, Switzerland).

### Data Sources

Data of participants enrolled in KIULARCO was captured in an electronic medical record system (OpenMRS) and was extracted for PWH (demographics, contraceptive use, alcohol consumption and tobacco use (smoked or chewed), and clinical information including CD4 and WHO stage). Open Data Kit was used to collect demographic data from partners without HIV, along with information on contraceptive use, circumcision status for men, sexual behavior, alcohol consumption and tobacco use (smoked or chewed), symptoms of sexually transmitted diseases, knowledge about HIV transmission and PrEP, reasons for accepting or declining PrEP, and adherence to PrEP among both partners (Supplement 1–4).

### Statistical Methods

A flowchart was used to describe the PrEP care continuum. PrEP uptake was assessed as the proportion of partners without HIV among serodifferent couples who initiated PrEP. Baseline characteristics were summarized using frequencies and percentages for categorical data and median and interquartile ranges (IQR) for continuous data for: (a) PWH with a partner without HIV or partner of unknown status, (b) PWH with a partner without HIV enrolled in PrEP, and (c) partners without HIV enrolled in the PrEP study.

Analyses were primarily descriptive, focusing on proportions at each step of the PrEP care continuum. Given the small number of couples enrolled in PrEP, we did not perform formal hypothesis testing, as statistical power would be too low to provide meaningful inference. Instead, differences between groups are reported descriptively to highlight potential trends and programmatic implications.

Viral load suppression was assessed as the proportion of PWH with viral suppression out of those enrolled into the PrEP study with their partner.

Data from follow-up questionnaires was compared to baseline to describe changes in behaviour of both partners over time, like number of sexual partners, frequency of unprotected sexual intercourse per month, use of family planning methods, pregnancies, symptoms of sexually transmitted diseases and alcohol consumption or tobacco use.

Adherence to PrEP was analysed at each visit through pill box return and pill count, and self-report of doses missed (proportions of pills taken). The safety of PrEP was assessed by registration of adverse events among partners without HIV on PrEP.

### Ethical Approval

We obtained ethical approval from the Ifakara Health Institute Institutional Review Board (IHI/IRB/No: 22-2020) and the National Institute for Medical Research of Tanzania (NIMR/HQ/R.8a/Vol.IX/3482). The KIULARCO study was approved by the Ifakara Health Institute Institutional Review Board (IHI/IRB/No16-2006) and the National Institute for Medical Research of Tanzania (NIMR/HQ/R.8a/Vol.IX/620).

Written informed consent for KIULARCO was obtained from all PWH aged ≥ 18 years, and from caregivers for adolescents aged < 18 years. In addition, written informed consent was obtained from all partners without HIV ≥ 18 years, and from caregivers for adolescents aged < 18 years.

## Results

### Study Population

**Fig. 1:**
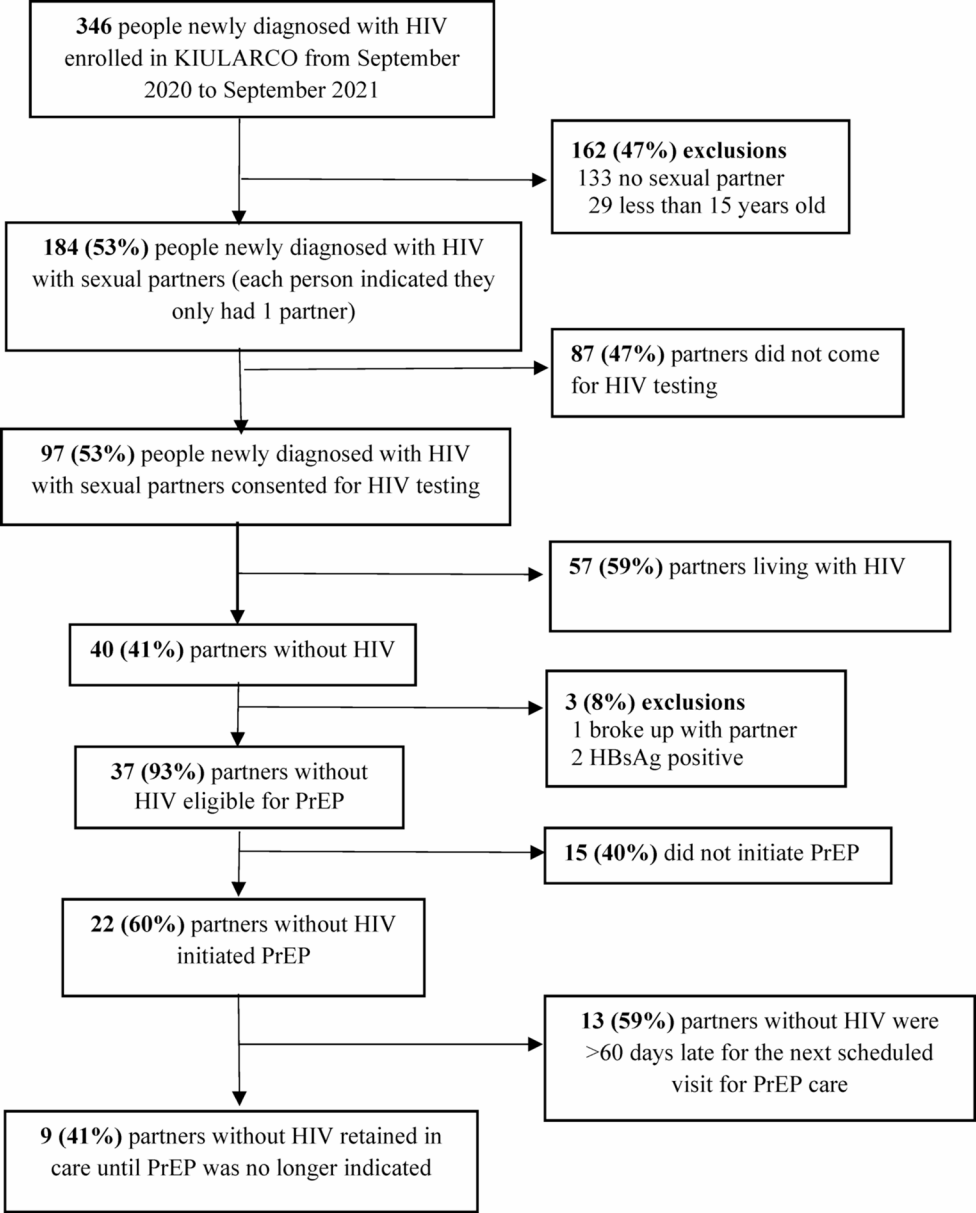
PrEP study flowchart. All percentages refer to denominator in above box

Out of 346 people newly diagnosed with HIV aged ≥ 15 years enrolled into KIULARCO from September 2020 to September 2021, 184 (53%) reported having a sexual partner (none had more than one). Of these, 87 (47%) did not bring their partner for HIV testing, while 97 (53%) partners consented to testing (Fig. 1). Among all those tested, 57 (59%) were seropositive, and 40 (41%) were partners without HIV. One couple was excluded due to a breakup after receiving their results, and two couples were excluded because the partner without HIV tested positive for HBsAg. Thus, 37 serodifferent couples were eligible for PrEP. Of those, 22 (60%) partners without HIV agreed to initiate PrEP.

127 PWH had a partner with a possible indication for PrEP: 40 had a seronegative status and 87 an untested partner. The median age was 38 years (IQR 29–47), 71 (56%) were female, the majority (102 (80%)) were married or cohabiting, and had a primary or secondary school education (109 (86%)). Only 77 (64%) had disclosed their HIV status, in 49 (64%) to their partner (Table 1). The majority of participants had an HIV WHO stage I/II (91 (75%)); 39 (37%) had a CD4 count ≥ 350 cells/µl.

Descriptively, the baseline characteristics of PWH who had a partner without HIV and enrolled in the PrEP study were largely similar to those of PWH with a partner whose HIV status was either negative or unknown. However, those with partner who initiated PrEP compared to all PWH with partners without HIV or unknown status were more likely to be married/cohabiting (21 (95%) vs. 102 (80%)) and in WHO stage I/II (19 (86%) vs. 91 (75%)).

**Table 1:** Baseline characteristics among people newly diagnosed with HIV and their partners

Characteristics	HIV positive with partner without HIV or unknown status, (*N* = 127)	HIV positive with partner without HIV initiated on PrEP, (*N* = 22)	Partner without HIV initiated on PrEP, (*N* = 22)
Socio-demographics			
Age, years, median (IQR)	38 (29–47)	38 (28–49)	37 (27–49)
Age, years, n (%)			
15–24	15 (12%)	3 (14%)	4 (18%)
25–34	34 (27%)	6 (27%)	7 (32%)
35–44	34 (27%)	4 (18%)	3 (14%)
≥ 45	44 (35%)	9 (41%)	8 (36%)
Gender, female, n (%)	71 (56%)	10 (46%)	12 (55%)
Marital status, n (%)			
Married/cohabiting	102 (80%)	21 (95%)	21 (95%)
Never married	2 (2%)	0 (0%)	0 (0%)
Separated/divorced/widowed	23 (18%)	1 (5%)	1 (5%)
Education, n (%)			
None	18 (14%)	4 (18%)	3 (14%)
Primary	91 (72%)	14 (64%)	15 (68%)
Secondary and above	18 (14%)	4 (18%)	4 (18%)
Occupation, n (%)			
Non farmer	36 (28%)	5 (22%)	5 (22%)
Farmer	91 (72%)	17 (77%)	17 (77%)
Disclosure of HIV status, n (%)			
Yes	77 (64%)	22 (100%)	NA
No	44 (36%)	0 (0%)	
Missing	6 (5%)	0 (0%)	
Disclosure to partner, n (%)	49/77(64%)	22 (100%)	NA
Smoking/alcohol habits, n (%)			
Chew tobacco	1/121 (1%)	0 (0%)	1/15 (7%)
Cigarettes, ever	4/121 (3%)	1 (5%)	1/15 (7%)
Alcohol	24/121 (20%)	4 (18%)	2/15 (13%)
Clinical			
HIV WHO stage^a^, n (%)			NA
I/II	91 (75%)	19 (86%)	
III/IV	30 (25%)	3 (14%)	
Missing	6 (5%)	0 (0%)	
CD4 count, cells/µl^a^, n (%)			NA
< 100	23 (22%)	4 (19%)	
100–349	43 (41%)	10 (48%)	
≥ 350	39 (37%)	7 (33%)	
Missing	22 (17%)	1 (5%)	

Results are number and column % of those with non-missing data, denominators indicator number of responses. Comparisons between groups are shown descriptively only; no statistical tests were performed due to the small sample size and risk of misleading inference

Defined as the date of enrolment in KIULARCO for PWH or date of enrolment in PrEP for partners without HIV

^a^HIV WHO stage and CD4 measurements closest to baseline within 6 months before and 1 month after

### Sexual Risk Behavior, Awareness of HIV Transmission and PrEP

Due to reluctance to answer questions about sexual risk behavior the questionnaires were amended during the study and thus data for these categories is missing. Of the newly enrolled PWH with a partner with possible PrEP indication, 36/65 (55%) answered that they lived in the same household with their partner, 32/65 (49%) had been in partnership for more than 3 years, and 23/65 (35%) had children (Table 2).

When comparing the PWH with a partner without HIV enrolled in the PrEP study to those with a partner without HIV or a partner of unknown status who did not enroll, 15/15 (100%) vs. 36/65 (55%) were living in the same household.

In the assessment of sexual risk behaviors, 43/65 (66%) had had sex in the previous month before enrolment, with 36 (84%) of these having unprotected sex. The majority of the participants (74/104 (71%)) were not using any family planning methods.

Among the people newly diagnosed with HIV with a partner who was either seronegative or had an unknown status, 93/101 (92%) were aware of how HIV is transmitted. Of those, 90/93 (97%) believed HIV was transmitted through sexual intercourse, 76/93 (82%) through needles, 48/93 (52%) via blood transfusions and 8/93 (8%) incorrectly thought it could be transmitted through kissing, touching or living in the same household (Table 2).

Among the partners without HIV who initiated PrEP, 17/20 (85%) of those who responded were aware of how HIV is transmitted. All (17/17 (100%)) reported sexual intercourse as a mode of transmission, while 12/17 (71%) reported through needles, 5/17 (29%) through blood transfusion and 6/17 (35%) incorrectly believed HIV could be transmitted through kissing (Table 2).

Only 9/101 (9%) of PWH and 2/22 (9%) of partners without HIV were aware of PrEP prior to participating in the study. Awareness of PrEP was slightly higher among PWH whose partners without HIV were enrolled in PrEP (3/22 (14%)). Most participants learned about PrEP from friends or media. Reasons for partners without HIV to start PrEP were primarily to prevent HIV infection (16/20 (80%)), followed by supporting their partner (12/20 (60%)), and for the ability to have children 3/20 (15%) (Table 2).

**Table 2:** Sexual risk behavior, awareness of HIV transmission and PrEP

Characteristics	HIV positive with partner without HIV or unknown status (*N* = 127)	HIV positive with partner without HIV initiated on PrEP, (*N* = 22)	Seronegative partner initiated on PrEP, (*N* = 22)
Behaviors			
Living in the same household, n (%)	36/65 (55%)	15/15 (100%)	15/15 (100%)
Time involved with partner, n (%)			
< 6 months	5/65 (8%)	1/15 (7%)	1/15 (7%)
6–12 months	8/65 (12%)	1/15 (7%)	1/15 (7%)
1–3 years	20/65 (31%)	4/15 (27%)	4/15 (27%)
> 3 years	32/65 (49%)	9/15 (60%)	9/15 (60%)
Child with partner, n (%)	23/65 (35%)	7/15 (47%)	7/15 (47%)
Sex with partner in last month, n (%)	43/65 (66%)	14/15 (93%)	14/15 (93%)
Unprotected sex in last month, n (%)	36/43 (84%)	11/14 (79%)	11/14 (79%)
Other sex partners, n (%)			
None	56/65 (86%)	15/15 (100%)	15/15 (100%)
1–2	6/65 (9%)	0/0 (0%)	0/0 (0%)
≥ 3	3/65 (5%)	0/0 (0%)	0/0 (0%)
Family planning, n (%)			
None	74/104 (71%)	17/22 (77%)	10/15 (67%)
Pills	1/71 (1%)	0/10 (0%)	0/10 (0%)
Implant	1/71 (1%)	0/10 (0%)	1/15 (7%)
IUD	1/71 (1%)	0/10 (0%)	1/15 (7%)
Condoms	11/38 (29%)	3/12 (25%)	3/10 (30%)
Depo	4/71 (6%)	0/10 (0%)	0/15 (0%)
Circumcision (Men), n (%)	28/38 (74%)	9/12 (75%)	8/10 (80%)
Pregnancy (women), n (%)	17/71 (24%)	4/10 (40%)	4/12 (33%)
Symptoms of STI, n (%)	17/101 (16%)	5/22 (33%)	1/20 (5%)
Awareness of HIV transmission, n (%)	93/101 (92%)	19/22 (86%)	17/20 (85%)
Sexual intercourse	90/93 (97%)	19/19 (100%)	17/17 (100%)
Kissing	1/93 (1%)	0/19 (0%)	1/17 (6%)
Touching	5/93 (5%)	0/19 (0%)	0/17 (0%)
Living same household	2/93 (2%)	0/19 (0%)	0/17 (0%)
Blood transfusion	48/93 (52%)	13/19 (68%)	5/17 (29%)
Needles	76/93 (82%)	18/19 (95%)	12/17 (71%)
Others (listed below)	20/93 (22%)	7/19 (37%)	3/17 (18%)
Awareness of PrEP, n (%)	9/101 (9%)	3/22 (14%)	2/20 (10%)
Media	4/9 (44%)	1/3 (33%)	0/2 (0%)
Brochure	1/9 (11%)	0/3 (0%)	0/2 (0%)
Health facility	2/9 (22%)	1/3 (33%)	1/2 (50%)
Friend	6/9 (67%)	2/3 (67%)	0/2 (0%)
Others	0/0 (0%)	0/0 (0%)	1/2 (50%)
Agree to use PrEP, n (%)	22/101 (22%)	22/22 (100%)	22/22 (100%)
Reasons to start PrEP, n (%)			
Prevent transmission			16/20 (80%)
Support partner			12/20 (60%)
Conceive			3/20 (15%)
Others			0/79 (0%)

Results are number and column % of those with non-missing data, denominators indicator number of responses. Comparisons between groups are descriptive only; no statistical tests were performed due to the small sample size and risk of misleading inference

*IUD*  intrauterine device, *STI* sexually transmitted infection

**Fig. 2:**
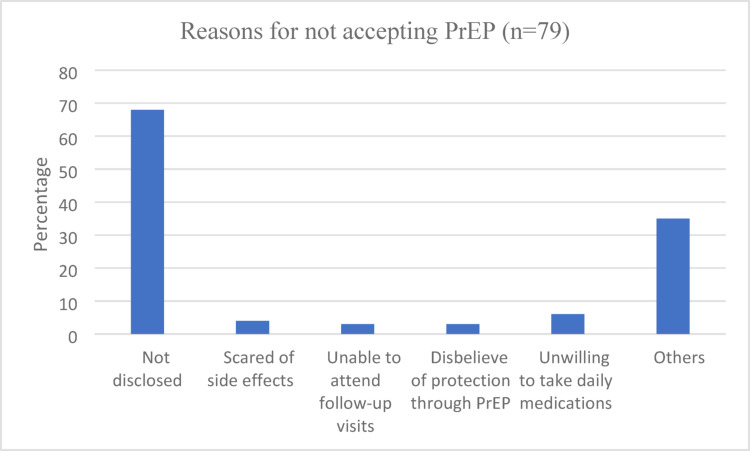
Reasons for not accepting PrEP (as indicated by PWH)

Reasons reported by PWH for declining PrEP for their partner included: not disclosing HIV status in 54/79 (68%), disbelief in protection from PrEP medication in 2/79 (3%), unwillingness to take daily medication in 5/79 (6%), being scared of side effects in 3/79 (4%) and others reasons 28/79 (35%) (Fig. 2). Of the other reasons, almost a third (8/28 (29%)) stated that their partner declined to get tested, other reasons were that the partner had travelled, was not actually the sexual partner or ended the relationship.

### Behaviors and Sexually Transmitted Diseases During Follow-Up

There was no change in the number of sexual partners, frequency of unprotected sexual intercourse, use of family planning methods, or change of alcohol consumption, cigarette smoking or tobacco chewing during the study period. During the follow-up visits, none of the participants reported new symptoms of sexually transmitted infections like discharge, genital ulcers or lower abdominal pain. Six partners on PrEP were tested for syphilis at the 6-month visit with 1 (17%) new positive result.

### Adherence, Side Effects and Completion of PrEP

84% of partners taking PrEP claimed to have never missed a dose. Most participants returned their pillboxes at follow-up visits (48/50 (96%)) and the number of unused pills indicated that at least 96% of dispensed pills had been taken as prescribed. There were six reported side effects, including stomach or digestive problems (2 cases), fatigue (1 case), nausea (2 cases) and sweating (1 case) in 50 visits during the study follow up among 3 (14%) seronegatives on PrEP, but no serious adverse events were reported. 9 of the 22 (41%) partners without HIV using PrEP were retained in care until no longer indicated. The remaining 13/22 (59%) were lost to follow up; 8 of these never returned after their baseline visit. There was no seroconversion among the 9 completing PrEP with a last HIV rapid test done 2 months after PrEP completion. Among them, 8/9 (89%) indicated they would recommend PrEP to other partners without HIV of people newly diagnosed with HIV. Among PWH with a partner without HIV, 21/22 (96%) were virally suppressed at 3 months. One participant was not suppressed after 3 months on ART with a VL of 2739 cp/ml at 3 months. After 6 months, the VL was repeated and not detectable. The partner remained on PrEP until viral suppression was confirmed.

## Discussion

This study highlights important gaps in the PrEP cascade in rural Tanzania. First of all, after asking newly diagnosed people with HIV to bring their partner for HIV testing, almost half of them (47%) did not bring their partner. Not disclosing their HIV status to the partner was a major reason why PrEP was not accepted. For the partners who were brought for testing, 59% were already positive. Furthermore, acceptance of PrEP in those who qualified was rather low with only 60%. There was almost no awareness of PrEP at the beginning of the study. Lastly, there was a high rate of lost to follow up leading to discontinuation of PrEP.

The first step of the PrEP care continuum is to identify people at risk of contracting HIV [10]. Since the majority of newly enrolled PWH into care at our clinic did not bring their partner for testing, this is the first gap in the prevention of HIV transmission in serodifferent couples in rural Tanzania. The reluctance to disclose their HIV status to a partner was very high with 68% saying that non-disclosure was the reason for not accepting PrEP. We believe that HIV-related stigma is the main reason for this, which is associated with non-HIV-status disclosure [24]. More efforts are thus needed in order to reduce HIV-related stigma. Results of a nested study in KIULARCO evaluating multiple measures to reduce stigma in newly diagnosed HIV-patients to assess a bundle of stigma-directed services (including video-assisted teaching, group support therapy and group health education through lay counselors, and appointment reminders through automated text messages as well as calls by a lay counselor) are currently under review [25].

Since over half of the partners who were brought for testing were positive, this underlines the importance of index testing as per guidelines, whereby exposed contacts of PWH are notified and offered an HIV test [21]. Yet the high proportion of partners testing HIV-positive in our study suggests missed opportunities for earlier diagnosis and prevention. This reflects limited community awareness of HIV status, low uptake of routine testing, missing awareness of PrEP, and ongoing barriers such as stigma. These findings highlight the need for earlier, community-based testing and strengthened prevention strategies for serodifferent couples.

In our study, acceptance of PrEP in serodifferent couples was 60%, thus rather low compared to the uptake of 97% as seen in the large implementation study done in Kenya and Uganda [13]. This is probably related to the low rate of awareness about PrEP, with only 9% of all newly diagnosed PWH aware of PrEP at baseline and 10% of partner without HIV. This highlights the need to increase community awareness. Radio and television spots were successfully used in the roll-out of the Kenyan PrEP program in 2017, reaching almost 12 million people, and another 350,000 through social media [26]. Other ways to increase awareness are through posters and education in clinics. Lay health providers and peer educators were effective in increasing knowledge about PrEP [27].

There was also a high rate of lost to follow up, resulting in discontinuation of PrEP. Reasons for this are unclear. Tracking through telephone calls was not effective. None of the those partners returned to care despite tracking them. Since PrEP follow-up was done at the clinic were PWH attend, again a reason might be HIV-related stigma causing reluctance to go to the clinic. Additionally, the rate of LTFU in PWH in this setting has been reported to be as high as 41% by 1 year after enrolment, with probable reasons apart from stigma being socioeconomic problems [28]. Interventions to reduce lost to follow up for both PWH and seronegative partners implemented in routine care are professional and lay counselling, tracking by telephone and contacting community health-care workers to follow-up on patients in case of missing visits. Evidence from sub-Saharan Africa shows that decentralizing PrEP delivery to community or pharmacy-based settings, integrating PrEP with reproductive health services, appointment reminders by SMS, or peer-support interventions improve continuity of care [9, 29, 30].

The standard “bridging” period of oral PrEP is 7 months [13]. That is, PrEP is initiated to the partner without HIV upon diagnosis and antiretroviral therapy (ART) initiation of the PWH and stopped 4 weeks after the PWH has a suppressed viral load, which in many African countries is routinely measured 6 months after starting antiretroviral therapy. With rollout of DTG-based ART, viral suppression is expected rapidly, with >85% having a suppressed VL after 1 month. We first measured VL after 3 months and at that point, 96% had achieved viral suppression. That led to a shortened PrEP bridging period of only 4 months for the partners. Only one participant had a high viral load, which was suppressed 3 months later and the partner continued PrEP for 7 months. So far, there are no studies comparing 4-month to 7-month PrEP as a bridging period. A barrier to PrEP has been reluctance to take medication for a longer time period, and take daily pills [31]. Routinely measuring VL after 3 instead of 6 months shortens the bridging period of PrEP in serodifferent couples. This might result in more people being willing to initiate PrEP, but this warrants further studies.

In this study, there was no increased report of symptoms of other sexually transmitted diseases as shown in other studies after PrEP initiation in men who have sex with men [32]. This is likely because participants stated to only have one partner, thus being at low risk for other sexual diseases. Higher risk behavior was noted for some (9/65 (14%)) of the PWH, having more than one sexual partner. These however did not have a partner enrolled into PrEP.

Our study has limitations. One limitation was the missing data from questionnaires. Participants were reluctant to answer questions about sexual behavior. To overcome this, we modified the questionnaire. As this was done halfway into the study, data are missing for the other half. Furthermore, the small sample size and single-center design of our study warrant caution in extending these findings to other settings. A further weakness is that within this study we could not properly compare a shortened 4-month bridging period against the 7-month standard of care, limiting conclusions about its effectiveness.

Finally, the small number of eligible couples precluded meaningful hypothesis testing. We therefore reported differences descriptively, which limits our ability to draw definitive conclusions about predictors of PrEP uptake.

In conclusion, our study highlights the gaps of the PrEP care cascade in a rural sub Saharan African setting. Lack of awareness and stigma remain significant barriers to PrEP uptake and HIV prevention in serodifferent couples.

This highlights the importance of differentiated service delivery models that are responsive to the realities of rural African settings, alongside with emerging options such as long-acting injectable PrEP.

## Supplementary Information

Below is the link to the electronic supplementary material.

Supplementary Material 1

Supplementary Material 2

Supplementary Material 3

Supplementary Material 4
