# Supplementary material for: PrEP Cascade and Barriers Among Serodifferent Couples in Rural Tanzania: A Prospective Study on Awareness, Uptake, Adherence, and Retention
Source: AIDS Behav. 2025 Nov 21;30(4):1229–38. doi: 10.1007/s10461-025-04957-8 (PMC13076417; doi:10.1007/s10461-025-04957-8)
Supplement: Supplementary file 1 — Supplementary Material 1 [file 10461_2025_4957_MOESM1_ESM.docx]

**PrEP cascade and barriers among serodifferent couples in rural Tanzania: A Prospective Study on Awareness, Uptake, Adherence, and Retention**

**Journal: *AIDS and Beahvior***

Anna Eichenberger [1], Lilian Moshi [2,3], James Okuma [4,5], Fiona Vanobberghen [4,5], Aloyce Sambuta [2,3], Olivia Kitau [2], Leila S. Matoy [2,3], Elizabeth Senkoro [2,9], Namvua Kimera [2,3], Mohamed Mbaruku [2,3], Jamali Siru [2,3], Raphael Magnolini [7,8], Tracy R. Glass [4,5], Maja Weisser [2,3,4,5,6]

[1] Department of Infectious Diseases, Bern University Hospital, Bern, Switzerland

[2] Ifakara Health Institute, Ifakara, Tanzania

[3] St. Francis Referral Hospital, Ifakara, Tanzania

[4] Swiss Tropical and Public Health Institute, Allschwil, Switzerland

[5] University of Basel, Basel, Switzerland

[6] Division of Infectious Diseases, University Hospital Basel, Switzerland

[7] Arud Centre for Addiction Medicine, Zurich, Switzerland

[8] Institute of Primary Care (IHAMZ), University of Zurich and University Hospital Zurich, Switzerland

[9] Kilimanjaro Christian Medical Centre, Moshi, Tanzania

**Corresponding Author**

Anna Eichenberger, MD, Department of Infectious Diseases, Bern University Hospital, Freiburgstrasse 20, 3010 Bern, Switzerland, [anna.eichenberger@insel.ch](mailto:anna.eichenberger@insel.ch); ORCID 0000-0001-9775-9424

**DODOSO LA AWALI KWA MWENZA MWENYE MAAMBUKIZI KWA AJILI YA TAFITI YA UTOAJI DAWA ZA VVU KAMA KINGA**

**PrEP Study Baseline Questionnaire for seropositive partner**

| TAREHE  Date | __: __: _____ (dd/mm/yyyy)) |
| --- | --- |
| Attendant Initials | _ _ _ |
| **NAMBA YA MGONJWA (NACP) NAMBA**  NACP No | …………. |
| Sex | 1 = Male / 2 = Female |
| Type of Visit | 1= Enrollment |
| Partner present at this visit | 0=No  1=Yes |
| Partner already tested for HIV | 0=No 1 = yes (then answer next question)  1 = Yes (then answer next question)  2=I do not know |
| Partner status | 0= Negative  1= Positive (then exclusion from this study, end of QN)  2= Unknown |
| Partner enrolled | 0= No  1= Yes (if yes put number below) |
| NAMBA YA PREP YA WENZA  Partner PrEP No (if enrolled) | ……….. |
| Age [≥](https://praxistipps.chip.de/latex-kleiner-gleich-und-groesser-gleich-schreiben-so-gehts_92369) 15 years | 0 =No (then exclusion from this study, end of QN)  1= Yes |
| Enrolled into KIUALRCO with written informed consent | 0 = No (then exclusion from this study, end of QN)  1= Yes |
|  |  |
|  |  |
| **Questions** | |
| 1a. Are you living in the same household with your partner?  JE UNAKAA NYUMBA MOJA NA MWENZA WAKO? | 0 = Hapana No  1= Ndio Yes |
| 1b. How long have you been in a relationship/involved with this partner?  JE, NI KWA MUDA GANI UMEKUWA KWENYE MAHUSIANO NA MWENZA HUYO? | 1= < 6 month  2= 6-12 months  3= 1-3 years  4= > 3 years |
| 1c. Do you have a child or children together with this partner?  JE, UMEZAA NA HUYO MWENZA? | 0 = Hapana No  1= Ndio Yes |
| 1d. Did you have sex with this partner in the last month? | 0 = Hapana No  1= Ndio Yes |
| 1e. If yes:  Did you have unprotected (condom less) sex with this partner in the last month? | 0 = Hapana No  1= Ndio Yes |
| 2a. KATIKA MWEZI ULIOPITA UMESHIRIKIANA NA WATU WENGINE WANGAPI KIMAPENZI?  How many other people did you have sex with in the last month?  *Hint: If a client does not want to answer, enter 99* | _ _ |
| 2b. Did you have unprotected (condom less) sex with this/these other partner/s in the last month?  *If question 2a. is 1 or more* | 0 = Hapana No  1= Ndio Yes |
| 2c.1 How long have you been in a relationship/involved with the other partner 1?  NI KWA MUDA GANI UMEKUWA KWENYE MAHUSIANO NA HUYO MWENZA WAKO WA PILI?  *If other sexual partner*  [≥](https://praxistipps.chip.de/latex-kleiner-gleich-und-groesser-gleich-schreiben-so-gehts_92369) *1 (question 2a)* | 1= < 6 month  2= 6-12 months  3= 1-3 years  4= > 3 years |
| 2d.1 Do you have a child or children together with this other partner 1?  JE, UMEZAA NA HUYO MWENZA WAKO WA PILI?  *If other sexual partner*  [≥](https://praxistipps.chip.de/latex-kleiner-gleich-und-groesser-gleich-schreiben-so-gehts_92369) *1 (question 2a)* | 0 = Hapana No  1= Ndio Yes |
| 2c.2 How long have you been in a relationship/involved with the other partner 2?  NI KWA MUDA GANI UMEKUWA KWENYE MAHUSIANO NA HUYO MWENZA WAKO WA TATU?  *If other sexual partner is*  [≥](https://praxistipps.chip.de/latex-kleiner-gleich-und-groesser-gleich-schreiben-so-gehts_92369) 2 *(question 2a)* | 1= < 6 month  2= 6-12 months  3= 1-3 years  4= > 3 years |
| 2d.2 Do you have a child or children together with this other partner 2?  JE, UMEZAA NA HUYO MWENZA WAKO WA TATU?  *If other sexual partner*  [≥](https://praxistipps.chip.de/latex-kleiner-gleich-und-groesser-gleich-schreiben-so-gehts_92369) *2 (question 2a)* | 0 = Hapana No  1= Ndio Yes |
| 2c.3 How long have you been in a relationship/involved with the other partner 3?  NI KWA MUDA GANI UMEKUWA KWENYE MAHUSIANO NA HUYO MWENZA WAKO WA NNE?  *If other sexual partner*  [≥](https://praxistipps.chip.de/latex-kleiner-gleich-und-groesser-gleich-schreiben-so-gehts_92369) *3 (question 2a)* | 1= < 6 month  2= 6-12 months  3= 1-3 years  4= > 3 years |
| 2d.3 Do you have a child or children together with this other partner 3?  JE, UMEZAA NA HUYO MWENZA WAKO WA NNE?    *If sexual partner*  [≥](https://praxistipps.chip.de/latex-kleiner-gleich-und-groesser-gleich-schreiben-so-gehts_92369) *3 (question 2a)* | 0 = Hapana No  1= Ndio Yes |
| 2c.4 How long have you been in a relationship/involved with the other partner 4?  NI KWA MUDA GANI UMEKUWA KWENYE MAHUSIANO NA HUYO MWENZA WAKO WA TANO?  *If other sexual partner*  [≥](https://praxistipps.chip.de/latex-kleiner-gleich-und-groesser-gleich-schreiben-so-gehts_92369) *4 (question 2a)* | 1= < 6 month  2= 6-12 months  3= 1-3 years  4= > 3 years |
| 2d.4 Do you have a child or children together with this other partner 4?  JE, UMEZAA NA HUYO MWENZA WAKO WA NNE?  *If other sexual partner*  [≥](https://praxistipps.chip.de/latex-kleiner-gleich-und-groesser-gleich-schreiben-so-gehts_92369) *4 (question 2a)* | 0 = Hapana No  1= Ndio Yes |
| 3.WANANAUME: NJIA YA UZAZI WA MPANGO/ NJIA ZA UKINGAJI WA MAGOJWA YA ZINAA  **Men:** Family planning methods / Prevention methods for STDs  *(For females in OpenMRS)*  *(tick all that apply)* | 1= HAKUNA none  2= VIDONGE pills  3 = SINDANO depot injection  4= VIPANDIKIZI implant  5= KITANZI IUD  6=KUFUNGA MIRIJA sterilization  7= KONDOM condom  8= KUKOJOA NJE withdrawal |
| 4. MWANAUME: UMEFANYIWA TOHARA  Men: Circumcision done | 0= Hapana No  1= Ndio Yes |
| 5. UCHUNGUZI WA MAGONJWA YA ZINAA  STI screening  (tick all that apply) | 1= Kutoka uchafu sehemu za siri  Urethral/ PV discharge  2= Kidonda sehemu ya siri  Genital Ulcer  3= Maumivu ya tumbo chini ya kitovu  Lower abdominal pain |
| 6. JE, UNAFAHAMU NI JINSI GANI VVU VINAWEZA KUSAMBWAZWA?  Are you aware how HIV can be transmitted?  *(if yes, tick all that apply)* | 0= Hapana No  1= Ndio Yes  (KAMA NDIO / if yes:)  1= KUJAMIIANA sexual intercourse  2= KUBUSIANA kissing  3= KUGUSANA touching  4= KUISHI NYUMBA MOJA living in same household  5= KUONGEZEWA DAMU blood transfusion  6= VITU VYENYE INCHA KALI needles  7= NYINGINE TAJA others, specify |
| 7. JE, UMEWAHI KUSIKIA KUHUSU DAWA KINGA ZA VVU?  Have you ever heard of pre-exposure prophylaxis?  KAMA NDIO, NI KWA NJIA GANI  *If yes, how (tick all that apply)* | 0= Hapana No  1= Ndio Yes  1= RADIO NA TELEVISHENI media  2= MAGAZETI newspaper  3= VIPEPERUSHI brochure  4= VITUO VYA AFYA health facility  5= RAFIKI friend  6= Group therapy session  7= NYINGINE, TAJA other, specify |
| 8. JE, MWENZA WAKO AMEKUBALI KUTUMIA DAWA KINGA ZA VVU (TDF/FTC)  Did serodiscordant partner agree to user PrEP (TDF/FTC)  KAMA HAPANA, SABABU  If no, why  *(tick all that apply)* | 0= Hapana No  1= Ndio Yes  1= Hajui kuhusu hali yangu ya maambukizi  Not disclosed  2= Ana hofu maudhi ya dawa  Scared of side effects  3= Hawezi kuja kwa maudhurio ya marudio  Unable to attend follow-up visits  4= Haamini kupata kinga kwa dawa kinga  Disbelieve of protection through PrEP  5= Ana hofu ya unyanyapaa  Fear of stigma  6= Hayupo tayari kutumia dawa za kila siku  Unwilling to take daily medication  7= Mengineyo, taja  Others, specify _____________________ |
