# Supplementary material for: PrEP Cascade and Barriers Among Serodifferent Couples in Rural Tanzania: A Prospective Study on Awareness, Uptake, Adherence, and Retention
Source: AIDS Behav. 2025 Nov 21;30(4):1229–38. doi: 10.1007/s10461-025-04957-8 (PMC13076417; doi:10.1007/s10461-025-04957-8)
Supplement: Supplementary file 2 — Supplementary Material 2 [file 10461_2025_4957_MOESM2_ESM.docx]

**PrEP cascade and barriers among serodifferent couples in rural Tanzania: A Prospective Study on Awareness, Uptake, Adherence, and Retention**

**Journal: *AIDS and Beahvior***

Anna Eichenberger [1], Lilian Moshi [2,3], James Okuma [4,5], Fiona Vanobberghen [4,5], Aloyce Sambuta [2,3], Olivia Kitau [2], Leila S. Matoy [2,3], Elizabeth Senkoro [2,9], Namvua Kimera [2,3], Mohamed Mbaruku [2,3], Jamali Siru [2,3], Raphael Magnolini [7,8], Tracy R. Glass [4,5], Maja Weisser [2,3,4,5,6]

[1] Department of Infectious Diseases, Bern University Hospital, Bern, Switzerland

[2] Ifakara Health Institute, Ifakara, Tanzania

[3] St. Francis Referral Hospital, Ifakara, Tanzania

[4] Swiss Tropical and Public Health Institute, Allschwil, Switzerland

[5] University of Basel, Basel, Switzerland

[6] Division of Infectious Diseases, University Hospital Basel, Switzerland

[7] Arud Centre for Addiction Medicine, Zurich, Switzerland

[8] Institute of Primary Care (IHAMZ), University of Zurich and University Hospital Zurich, Switzerland

[9] Kilimanjaro Christian Medical Centre, Moshi, Tanzania

**Corresponding Author**

Anna Eichenberger, MD, Department of Infectious Diseases, Bern University Hospital, Freiburgstrasse 20, 3010 Bern, Switzerland, [anna.eichenberger@insel.ch](mailto:anna.eichenberger@insel.ch); ORCID 0000-0001-9775-9424

**DODOSO LA AWALI KWA MWENZA KWA AJILI YA TAFITI YA UTOAJI DAWA ZA VVU KAMA KINGA**

**PrEP Study Baseline Questionnaire for seronegative partner**

| TAREHE  Date | | __ : __: _____ (dd/mm/yyyy) |
| --- | --- | --- |
| Attendant Initials | | _ _ _ |
| PrEP NAMBA  PrEP No | | …………. |
| Sex | | 1 = Male / 2 = Female |
| Type of Visit | | 1= Enrollment |
| NAMBA NACP YA WENZA  Partner NACP No | | ……….. |
| Age [≥](https://praxistipps.chip.de/latex-kleiner-gleich-und-groesser-gleich-schreiben-so-gehts_92369) 15 years  Umri [≥](https://praxistipps.chip.de/latex-kleiner-gleich-und-groesser-gleich-schreiben-so-gehts_92369) miaka 15 | | 0 = No (then exclusion from this study, end of QN)  1= Yes |
| HIV Rapid Test result  Majibu ya kipimo cha VVU | | 0= Negative Hasi  1= Positive Chanya (then exclusion from this study, end of QN)  2= Indeterminant Tata |
| Where was the first HIV test performed  Kipimo cha kwanza cha VVU kilifanyika wapi | | 1= at home (home-based testing)  Nyumbani (upimaji wa majumbani)  2= at CDCI  CDCI  3= at other CTC  CTC nyingine  4= other, specify_________  Pengine, taja |
| Partner newly diagnosed with HIV, enrolled into KIULARCO  Mwenza aliyegundulika na VVU, amejiunga na KIULARCO | | 0 = Hapana No(then exclusion from this study, end of QN)  1= Ndio Yes |
| Sexually active with HIV-infected partner and intention to remain a couple  Anashiriki ngono na mwenza mwenye VVU na yupo tayari kubakia kwenye mahusiano | | 0= Hapana No (then exclusion from this study, end of QN)  1= Yes Ndio |
| HBsAg positive | | 0 =No 1= Yes(then exclusion from this study, end of QN) |
| eGFR < 60ml/min | | 0 = No 1= Yes (then exclusion from this study, end of QN) |
| Signs of acute HIV infection NOT explained by other illness (fever, fatigue, rash, headache, pharyngitis, lymphadenopathy, myalgia, night sweats, diarrhea) | | 0 = No  1= Yes |
| If answer to above question is yes, do a viral load Gene expert | | 1= VL Gene expert not detectable (include into study)  2= VL positive (confirm with second sample, exclude from study, end of QN) |
| < 18 years and still attending school | | 0= No 1= Yes (then exclusion from this study, end of QN) |
| Inclusion/Exclusion Criteria approved by Clinicians | | 0 = No  1= Yes |
|  |  |  |
| **Questions** | | |
| 1a. Are you living in the same household with your partner?  JE UNAKAA NYUMBA MOJA NA MWENZA WAKO? | | 0 = Hapana No  1= Ndio Yes |
| 1b. How long have you been in a relationship/involved with this partner?  JE, NI KWA MUDA GANI UMEKUWA KWENYE MAHUSIANO NA MWENZA HUYO? | | 1= < 6 month  2= 6-12 months  3= 1-3 years  4= > 3 years |
| 1c. Do you have a child or children together with this partner?  JE, UMEZAA NA HUYO MWENZA? | | 0 = Hapana No  1= Ndio Yes |
| 1d. Did you have sex with this partner in the last month? | | 0 = Hapana No  1= Ndio Yes |
| 1e. If yes:  Did you have unprotected (condom less) sex with this partner in the last month? | | 0 = Hapana No  1= Ndio Yes |
| 2a. KATIKA MWEZI ULIOPITA UMESHIRIKIANA NA WATU WENGINE WANGAPI KIMAPENZI?  How many other people did you have sex with in the last month?  *Hint: If a client does not want to answer, enter 99* | | _ _ |
| 2b. Did you have unprotected (condom less) sex with this/these other partner/s in the last month?  *If question 2a. is 1 or more* | | 0 = Hapana No  1= Ndio Yes |
| 2c.1 How long have you been in a relationship/involved with the other partner 1?  NI KWA MUDA GANI UMEKUWA KWENYE MAHUSIANO NA HUYO MWENZA WAKO WA PILI?  *If other sexual partner*  [≥](https://praxistipps.chip.de/latex-kleiner-gleich-und-groesser-gleich-schreiben-so-gehts_92369) *1 (question 2a)* | | 1= < 6 month  2= 6-12 months  3= 1-3 years  4= > 3 years |
| 2d.1 Do you have a child or children together with this other partner 1?  JE, UMEZAA NA HUYO MWENZA WAKO WA PILI?  *If other sexual partner*  [≥](https://praxistipps.chip.de/latex-kleiner-gleich-und-groesser-gleich-schreiben-so-gehts_92369) *1 (question 2a)* | | 0 = Hapana No  1= Ndio Yes |
| 2c.2 How long have you been in a relationship/involved with the other partner 2?  NI KWA MUDA GANI UMEKUWA KWENYE MAHUSIANO NA HUYO MWENZA WAKO WA TATU?  *If other sexual partner is*  [≥](https://praxistipps.chip.de/latex-kleiner-gleich-und-groesser-gleich-schreiben-so-gehts_92369) 2 *(question 2a)* | | 1= < 6 month  2= 6-12 months  3= 1-3 years  4= > 3 years |
| 2d.2 Do you have a child or children together with this other partner 2?  JE, UMEZAA NA HUYO MWENZA WAKO WA TATU?  *If other sexual partner*  [≥](https://praxistipps.chip.de/latex-kleiner-gleich-und-groesser-gleich-schreiben-so-gehts_92369) *2 (question 2a)* | | 0 = Hapana No  1= Ndio Yes |
| 2c.3 How long have you been in a relationship/involved with the other partner 3?  NI KWA MUDA GANI UMEKUWA KWENYE MAHUSIANO NA HUYO MWENZA WAKO WA NNE?  *If other sexual partner*  [≥](https://praxistipps.chip.de/latex-kleiner-gleich-und-groesser-gleich-schreiben-so-gehts_92369) *3 (question 2a)* | | 1= < 6 month  2= 6-12 months  3= 1-3 years  4= > 3 years |
| 2d.3 Do you have a child or children together with this other partner 3?  JE, UMEZAA NA HUYO MWENZA WAKO WA NNE?    *If sexual partner*  [≥](https://praxistipps.chip.de/latex-kleiner-gleich-und-groesser-gleich-schreiben-so-gehts_92369) *3 (question 2a)* | | 0 = Hapana No  1= Ndio Yes |
| 2c.4 How long have you been in a relationship/involved with the other partner 4?  NI KWA MUDA GANI UMEKUWA KWENYE MAHUSIANO NA HUYO MWENZA WAKO WA TANO?  *If other sexual partner*  [≥](https://praxistipps.chip.de/latex-kleiner-gleich-und-groesser-gleich-schreiben-so-gehts_92369) *4 (question 2a)* | | 1= < 6 month  2= 6-12 months  3= 1-3 years  4= > 3 years |
| 2d.4 Do you have a child or children together with this other partner 4?  JE, UMEZAA NA HUYO MWENZA WAKO WA NNE?  *If other sexual partner*  [≥](https://praxistipps.chip.de/latex-kleiner-gleich-und-groesser-gleich-schreiben-so-gehts_92369) *4 (question 2a)* | | 0 = Hapana No  1= Ndio Yes |
| 3. NJIA YA UZAZI WA MPANGO/ NJIA ZA UKINGAJI WA MAGOJWA YA ZINAA  Family planning methods / Prevention methods for STIs  (tick all that apply) | | 1= HAKUNA none  2= VIDONGE pills  3 = SINDANO depot injection  4= VIPANDIKIZI implant  5= KITANZI IUD  6=KUFUNGA MIRIJA sterilization  7= KONDOM condom  8= KUKOJOA NJE withdrawal |
| 4. HALI YA UJAZITO  Women: Pregnancy | | 0 = Hapana / 1= Ndio  0 = No / 1= Yes  If yes expected date of delivery  __ : __: _____ (day:month:year) |
| 5. MWANAUME: UMEFANYIWA TOHARA  Men: Circumcision done | | 0= Hapana No  1= Ndio Yes |
| 6 a. Kutafuna tumbaku / Chewing tobacco  Kuvuta sigara / Smoking  Mwaka ulioanza kuvuta / Year started smoking  Mwaka uliocha kuvuta / Year stopped smoking  Wastani wa idadi ya vipande vya sigara kwa siku / Average number of cigarettes smoked per day  b. Kwa sasa unatumia pombe Currently consume alcohol  Aina ya pombe Type of alcohol (select all that apply)  Matumizi kwa pombe kwa siku Daily alcohol consumption  Idadi kwa siku Number of standard drinks per day  Kiasi kwa wiki Weekly alcohol consumption  Idadi kwa siku Number of standard drinks per day | | 1 = Never Kamwe / 2= Current sasa / 3=Stopped Nimeacha  1 = Never Kamwe / 2= Current sasa / 3=Stopped nimeacha  ____  ____  ____  0 = Hapana No / 1= Ndio Yes  1 = Beer Bia 2= Liquor pombe 3=Nyingine Other  0 = Hapana No / 1= Ndio Yes  ____  0 = Hapana No /1= Ndio Yes  ____ |
| 7. UCHUNGUZI WA MAGONJWA YA ZINAA  STI screening  (tick all that apply) | | 1= Kutoka uchafu sehemu za siri  Urethral/ PV discharge  2= Kidonda sehemu ya siri  Genital Ulcer  3= Maumivu ya tumbo chini ya kitovu  Lower abdominal pain |
| 8. MATUMIZI YA AWALI YA ARV  Prior exposure to ART  *(tick all that apply)* | | 1= PEP  2= PREP  3= PMTCT  4= MENGINEYO other  5= HAKUNA none |
| 9. JE, UNAFAHAMU NI JINSI GANI VVU VINAWEZA KUSAMBWAZWA  Are you aware how HIV can be transmitted?  *(if yes, tick all that apply)* | | 0= Hapana No  1= Ndio Yes  (KAMA NDIO / if yes:)  1= KUJAMIIANA sexual intercourse  2= KUBUSIANA kissing  3= KUGUSANA touching  4= KUISHI NYUMBA MOJA living in same household  5= KUONGEZEWA DAMU blood transfusion  6= VITU VYENYE INCHA KALI needles  7= NYINGINE TAJA others, specify |
| 10. JE, UMEWAHI KUSIKIA KUHUSU DAWA KINGA ZA VVU?  Have you ever heard of pre exposure prophylaxis before attending this clinic?  KAMA NDIO, NI KWA NJIA GANI  *If yes, how (tick all that apply)* | | 0 = Hapana No  1= Ndio Yes  1= RADIO NA TELEVISHENI media  2= MAGAZETI newspaper  3= VIPEPERUSHI brochure  4= VITUO VYA AFYA Health facility  5= RAFIKI friend  6= Group therapy session  7= MENGINEYO other |
| 11. KWANINI UMEKUBALI KUTUMIA DAWA KINGA ZA VVU (TDF/FTC)  Why did you agree to use PrEP (TDF/FTC)  *(tick all that apply)* | | 1= Kuzui maambukizi  To prevent transmission  2= Kumuunga mkono mwenza  To support partner  3= Niwe na uwezo wa kupata watoto  To be able to have children  4= Mengineyo, taja ………  Others, specify |
| 12. Date of next visit | | __ : __: _____ (dd/mm/yyyy) |
