# Supplementary material for: PrEP Cascade and Barriers Among Serodifferent Couples in Rural Tanzania: A Prospective Study on Awareness, Uptake, Adherence, and Retention
Source: AIDS Behav. 2025 Nov 21;30(4):1229–38. doi: 10.1007/s10461-025-04957-8 (PMC13076417; doi:10.1007/s10461-025-04957-8)
Supplement: Supplementary file 3 — Supplementary Material 3 [file 10461_2025_4957_MOESM3_ESM.docx]

**PrEP cascade and barriers among serodifferent couples in rural Tanzania: A Prospective Study on Awareness, Uptake, Adherence, and Retention**

**Journal: *AIDS and Beahvior***

Anna Eichenberger [1], Lilian Moshi [2,3], James Okuma [4,5], Fiona Vanobberghen [4,5], Aloyce Sambuta [2,3], Olivia Kitau [2], Leila S. Matoy [2,3], Elizabeth Senkoro [2,9], Namvua Kimera [2,3], Mohamed Mbaruku [2,3], Jamali Siru [2,3], Raphael Magnolini [7,8], Tracy R. Glass [4,5], Maja Weisser [2,3,4,5,6]

[1] Department of Infectious Diseases, Bern University Hospital, Bern, Switzerland

[2] Ifakara Health Institute, Ifakara, Tanzania

[3] St. Francis Referral Hospital, Ifakara, Tanzania

[4] Swiss Tropical and Public Health Institute, Allschwil, Switzerland

[5] University of Basel, Basel, Switzerland

[6] Division of Infectious Diseases, University Hospital Basel, Switzerland

[7] Arud Centre for Addiction Medicine, Zurich, Switzerland

[8] Institute of Primary Care (IHAMZ), University of Zurich and University Hospital Zurich, Switzerland

[9] Kilimanjaro Christian Medical Centre, Moshi, Tanzania

**Corresponding Author**

Anna Eichenberger, MD, Department of Infectious Diseases, Bern University Hospital, Freiburgstrasse 20, 3010 Bern, Switzerland, [anna.eichenberger@insel.ch](mailto:anna.eichenberger@insel.ch); ORCID 0000-0001-9775-9424

**DODOSO LA HUDHURIO LA MARUDIO KWA MWENZA MWENYE MAAMBUKIZI KWA AJILI YA TAFITI YA UTOAJI DAWA ZA VVU KAMA KINGA**

**PrEP Study Follow-Up Questionnaire for seropositive partner**

| TAREHE  Date | __ : __: _____ (dd/mm/yyyy)) |
| --- | --- |
| Attendant Initials | _ _ _ |
| **NAMBA YA MGONJWA (NACP) NAMBA**  NACP No | …………. |
| Sex | 1 = Male / 2 = Female |
| Type of Visit (Follow Up timepoint)  *(Tick one)* | 2 = week 4 (day 28 (21-42))  3 = week 8 (day 56 (49-70))  4 = week 12 (day 84 (77-98))  5 = week 16 (day 112 (105-126))  6= week 24 (day 168 (161-182))  7= week 28 (day 196 (189-210))  8= week 36 (day 252 (245-266))  9 = Unscheduled |
| NAMBA YA PREP YA WENZA  Partner PrEP No | ……….. |
|  |  |
| **Questions** | |
| 1. IDADI YA WENZA  Number of Partners | _ _ |
| 2.a. NGONO ZEMBE NDANI YA MIEZI SITA ILIYOPITA  Unprotected sex in last month | 0 = Hapana / 1= Ndio  0= No / 1= Yes |
| 2b. MARA NGAPI UMESHIRIKI NGONO BILA KINGA NA MWENZA WAKO ALIYE KWENYE DAWA KINGA KWA KIPINDI CHA MWEZI MMOJA ULIOPITA?  How many times did you have unprotected sex with your partner (taking PreP) in the last month | 1= 1-2 times per month Mara 1-2 kwa mwezi  2= 2-5 times per month Mara 2-5 kwa mwezi  3= > 5 times per month Zaidi ya mara 5 kwa mwezi |
| 3. WANANAUME: NJIA YA UZAZI WA MPANGO/ NJIA ZA UKINGAJI WA MAGOJWA YA ZINAA  **Men:** Family planning methods / Prevention methods for STDs  *(For females in OpenMRS)* | 1= HAKUNA none  2= VIDONGE pills  3 = SINDANO depot injection  4= VIPANDIKIZI implant  5= KITANZI IUD  6=KUFUNGA MIRIJA sterilization  7= KONDOM condom  8= KUKOJOA NJE withdrawal |
| 4. UCHUNGUZI WA MAGONJWA YA ZINAA  STI screening  (tick all that apply) | 1= Kutoka uchafu sehemu za siri  Urethral/ PV discharge  2= Kidonda sehemu ya siri  Genital Ulcer  3= Maumivu ya tumbo chini ya kitovu  Lower abdominal pain |
| 5. Is this the patient’s last visit? (If yes, proceed to question 6) | 0= No  1= Yes |
| 6. On a scale form 0-10 (0=not likely, 10= very likely), how likely are you to recommend PrEP to other partners of newly infected people living with HIV?  Kati ya 0-10 (0= sitapendekeza, 10=nitapendekeza), ni kwa kiasi gani ungependekeza dawa kinga ya maambukizi ya VVU kwa wenza wa watu waliogundulika kuishi na virusi vya ukimwi. | _________ |
