# Supplementary material for: PrEP Cascade and Barriers Among Serodifferent Couples in Rural Tanzania: A Prospective Study on Awareness, Uptake, Adherence, and Retention
Source: AIDS Behav. 2025 Nov 21;30(4):1229–38. doi: 10.1007/s10461-025-04957-8 (PMC13076417; doi:10.1007/s10461-025-04957-8)

**PrEP cascade and barriers among serodifferent couples in rural Tanzania: A Prospective Study on Awareness, Uptake, Adherence, and Retention**

**Journal: *AIDS and Beahvior***

Anna Eichenberger [1], Lilian Moshi [2,3], James Okuma [4,5], Fiona Vanobberghen [4,5], Aloyce Sambuta [2,3], Olivia Kitau [2], Leila S. Matoy [2,3], Elizabeth Senkoro [2,9], Namvua Kimera [2,3], Mohamed Mbaruku [2,3], Jamali Siru [2,3], Raphael Magnolini [7,8], Tracy R. Glass [4,5], Maja Weisser [2,3,4,5,6]

[1] Department of Infectious Diseases, Bern University Hospital, Bern, Switzerland

[2] Ifakara Health Institute, Ifakara, Tanzania

[3] St. Francis Referral Hospital, Ifakara, Tanzania

[4] Swiss Tropical and Public Health Institute, Allschwil, Switzerland

[5] University of Basel, Basel, Switzerland

[6] Division of Infectious Diseases, University Hospital Basel, Switzerland

[7] Arud Centre for Addiction Medicine, Zurich, Switzerland

[8] Institute of Primary Care (IHAMZ), University of Zurich and University Hospital Zurich, Switzerland

[9] Kilimanjaro Christian Medical Centre, Moshi, Tanzania

**Corresponding Author**

Anna Eichenberger, MD, Department of Infectious Diseases, Bern University Hospital, Freiburgstrasse 20, 3010 Bern, Switzerland, [anna.eichenberger@insel.ch](mailto:anna.eichenberger@insel.ch); ORCID 0000-0001-9775-9424

**DODOSO LA HUDHURIO LA MARUDIOKWA MWENZA KWA AJILI YA TAFITI YA UTOAJI DAWA ZA VVU KAMA KINGA**

**PrEP Study Follow-Up Questionnaire for seronegative partner**

| TAREHE  Date | __ : __: _____ (dd/mm/yyyy) |
| --- | --- |
| Attendant Initials | _ _ _ |
| PrEP NAMBA  PrEP No | …………. |
| Sex | 1 = Male / 2 = Female |
| Type of Visit (Follow-Up timepoint)  *(Tick one)* | 2 = week 4 (day 28 (21-42))  3 = week 8 (day 56 (49-70))  4 = week 12 (day 84 (77-98))  5 = week 16 (day 112 (105-126))  6= week 24 (day 168 (161-182))  7= week 28 (day 196 (189-210))  8= week 36 (day 252 (245-266))  9 = Unscheduled |
| NAMBA NACP YA WENZA  Partner NACP No | ……….. |
| HIV Rapid Test result | 0 = Negative  1= Positive (if positive complete QN, send to clinician)  2=Indeterminant |
|  |  |
| **Questions** | |
| 1. IDADI YA WENZA  Number of Partners | _ _ |
| 2.a. NGONO ZEMBE NDANI YA MIEZI SITA ILIYOPITA  Unprotected sex in last month | 0 = Hapana/ 1= Ndio  0 =No / 1= Yes |
| 2b. MARA NGAPI UMESHIRIKI NGONO BILA KINGA NA MWENZA WAKO ALIYE KWENYE DAWA KINGA KWA KIPINDI CHA MWEZI MMOJA ULIOPITA?  How many times did you have unprotected sex with your partner (taking ART) in the last month | 1= 1-2 times per month / Mara 1-2 kwa mwezi  2= 2-5 times per month / Mara 2-5 kwa mwezi  3= > 5 times per month / Zaidi ya mara 5 kwa mwezi |
| 3. NJIA YA UZAZI WA MPANGO/ NJIA ZA UKINGAJI WA MAGOJWA YA ZINAA  Family planning methods / Prevention methods for STDs  (tick all that apply) | 1= HAKUNA none  2= VIDONGE pills  3 = SINDANO depot injection  4= VIPANDIKIZI implant  5= KITANZI IUD  6=KUFUNGA MIRIJA sterilization  7= KONDOM condom  8= KUKOJOA NJE withdrawal |
| 4. HALI YA UJAZITO TANGU UDHURUO LA MWISHO  Women: Pregnancy since last visit | 0=Hapana / 1= Ndio  0 = No / 1= Yes  If yes expected date of delivery / Kama ndiyo tarehe anayotazamia kujifungua  __ : __: _____ (day:month:year) |
| Is this the stop date of Truvada | 0=No1=Yes  If yes, proceed with next question |
| result of Pregnancy test (urine) | 0= Negative  1= Positive |
| 5 MATUMIZI YA VILEVI  Toxic habit  a. Kutafuna tumbaku / Chewing tobacco  Kuvuta sigara / Smoking  Mwaka ulioanza kuvuta / Year started smoking  Mwaka uliocha kuvuta / Year stopped smoking  Wastani wa idadi ya vipande vya sigara kwa siku / Average number of cigarettes smoked per day  b. Kwa sasa unatumia pombe Currently consume alcohol  Aina ya pombe Type of alcohol (select all that apply)  Matumizi kwa pombe kwa siku Daily alcohol consumption  Idadi kwa siku Number of standard drinks per day  Kiasi kwa wiki Weekly alcohol consumption  Idadi kwa siku Number of standard drinks per day | 1 = Never Kamwe / 2= Current sasa / 3=Stopped Nimeacha  1 = Never Kamwe / 2= Current sasa / 3=Stopped nimeacha  ____  ____  ____  0 = Hapana No / 1= Ndio Yes  1 = Beer Bia 2= Liquor pombe 3=Nyingine Other  0 = Hapana No / 1= Ndio Yes  ____  0 = Hapana No /1= Ndio Yes |
| 6. UCHUNGUZI WA MAGONJWA YA ZINAA  STI screening  (tick all that apply) | 1= Kutoka uchafu sehemu za siri  Urethral/ PV discharge  2= Kidonda sehemu ya siri  Genital Ulcer  3= Maumivu ya tumbo chini ya kitovu  Lower abdominal pain |
| 7. HALI YA UFUASI WA DAWA KINGA ZA VVU  PrEP adherence follow-up status   1. UMEKUJA NA KIFUNGASHIO CHA DAWA   Pill box return | 0= Hapana / No  1= Ndio / Yes |
| 1. IDADI YA SIKU ZA DAWA ZILIZOBAKI?   Number of days of pills remaining | _ _ |
| 1. JE, NI MARA NGAPI UMEKOSA KUNYWA DAWA NDANI YA WIKI 4 ZILIZOPITA?   How many times did you miss a dose in the previous 4 weeks? | 1 = Never  2 = Once a month  3 = Once every 2 weeks  4 = Once a week  5= Daily |
| 1. JE, UMEKOSA ZAIDI YA DOZI MBILI KWEYE MSTARI/SAFU?   Did you miss >= 2 doses in a row?  SABABU  Why (tick all that apply) | 0 = Hapana No  1=Ndio Yes no  If yes  1= NILISAHAU Forgot  2= NILISAFIRI travelled  3= DAWA ZILIISHA ran out of medication  4= NILIENDA KWENEYE MAAZISHI attended funeral  5= SIAMINI TENA DAWA KINGA ZA VVU don’t belive in PrEP anymore  6= NIMEACHANA NA MWENZA broke up with partner  7= NYINGINE, TAJA others, specify ............ |
| 8. UMEPATA MADHARA YEYOTE? Did you experience any side effects? | 0 =Hapana No  1= Ndio Yes |
| 9. KAMA NDIO, UMEPATA MADHARA GANI?  If yes, Which side effects? (tick all that apply) | 1= MAUMIVU YA KICHWA Headache  2= MAUMIVU YA TUMBO AU MATATIZO CHAKULA? Stomach/digestive problems  3= UPELE Rash  4= UCHOVU Fatigue  5= HALI YA KUONA KIZUNGUZUNGU Vertigo  6= MATATIZO YA KULALA Sleep problems  7= NYINGINE, TAJA others, specify ............ |
| 10. Date of next visit | __ : __: _____ (dd/mm/yyyy) |
| 11. If this is your last visit, please answer the following questions:  On a scale form 0-10 (0=not likely, 10= very likely), how likely are you to recommend PrEP to other partners of newly infected people living with HIV?  Kati ya 0-10 (0= sitapendekeza, 10=nitapendekeza), ni kwa kiasi gani ungependekeza dawa kinga ya maambukizi ya VVU kwa wenza wa watu wanaogundulika kuishi na virusi vya ukimwi? | ­­______ |
| On FU week 4 and 24, Depression Assessment is done: |  |


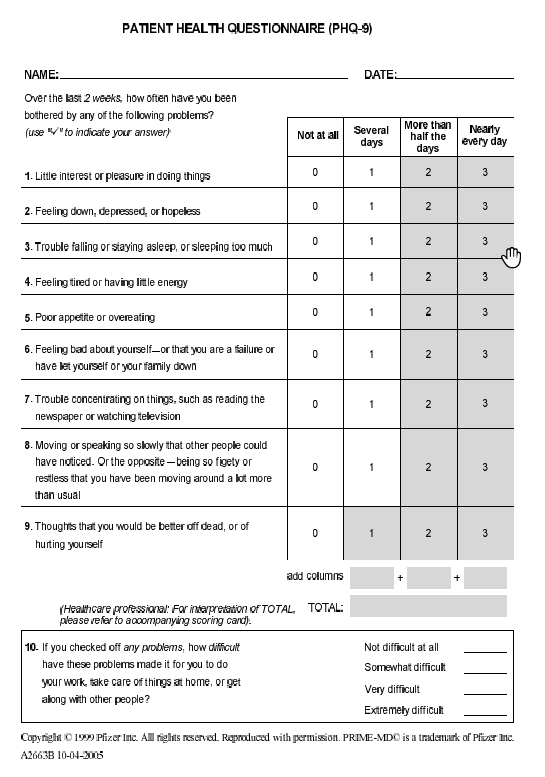


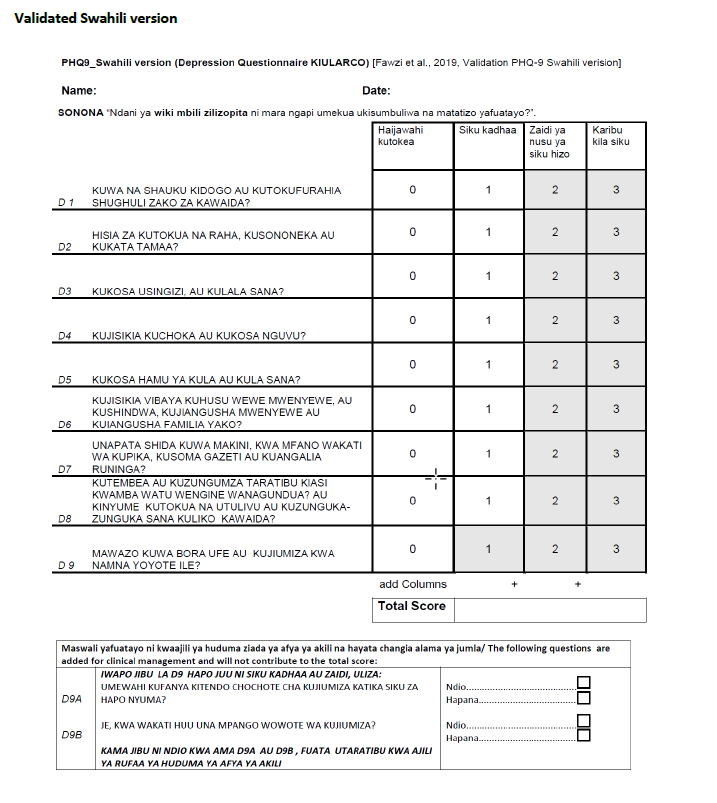

Supplement: Supplementary file 4 — Supplementary Material 4 [file 10461_2025_4957_MOESM4_ESM.docx]
